# Population-Based Estimate of Melioidosis, Kenya

**DOI:** 10.3201/eid2505.180545

**Published:** 2019-05

**Authors:** Esther M. Muthumbi, Nicola C. Gordon, George Mochamah, Sammy Nyongesa, Emily Odipo, Salim Mwarumba, Neema Mturi, Anthony O. Etyang, David A.B. Dance, J. Anthony G. Scott, Susan C. Morpeth

**Affiliations:** KEMRI–Wellcome Trust Research Programme, Kilifi, Kenya (E.M. Muthumbi, N.C. Gordon, G. Mochamah, S. Nyongesa, E. Odipo, S. Mwarumba, N. Mturi, A.O. Etyang, J.A.G. Scott, S.C. Morpeth);; London School of Hygiene & Tropical Medicine, London, UK (N.C. Gordon, D.A.B. Dance, J.A.G. Scott, S.C. Morpeth);; Lao-Oxford-Mahosot Hospital–Wellcome Trust Research Unit, Vientiane, Laos (D.A.B. Dance);; University of Oxford, Oxford, UK (D.A.B. Dance, J.A.G. Scott, S.C. Morpeth)

**Keywords:** *Burkholderia pseudomallei*, incidence, Kenya, melioidosis, surveillance, PCR, misidentification, bacteria

## Abstract

Melioidosis is thought to be endemic, although underdiagnosed, in Africa. We identified 5 autochthonous cases of *Burkholderia pseudomallei* infection in a case series in Kenya. Incidence of *B. pseudomallei* bacteremia in Kenya’s Kilifi County is low, at 1.5 cases per million person-years, but this result might be an underestimate.

*Burkholderia pseudomallei*, the causative agent of melioidosis, is a gram-negative bacillus endemic particularly in northern Australia and South and Southeast Asia. Worldwide, *B. pseudomallei* causes ≈165,000 cases of disease and ≈89,000 deaths annually (*1*). The presence of *B. pseudomallei* in Africa has been demonstrated by sporadic cases of melioidosis reported in travelers returning from countries including Kenya (*2*). Indigenous culture-confirmed cases have been reported in only 4 countries in Africa, mainly from research centers with diagnostic laboratory facilities (*3*). 

The first case of melioidosis linked to Kenya was diagnosed in 1982 in a tourist from Denmark who had visited Nyali (an area of Mombasa City), ≈50 km south of the town of Kilifi (*2*). Follow-up clinical surveillance in Nairobi and environmental surveillance from other regions in Kenya yielded no *B. pseudomallei* isolates (*4*)*.* However, growing concerns over possible underestimation of the disease in potentially endemic areas, including in tropical Africa, have led to calls for improved surveillance (*5*).

In 2010, at Kilifi County Hospital (KCH), we isolated *B. pseudomallei* from the blood culture of a 3-year-old child after a near-drowning accident in a seasonal river. The identity of the isolate was confirmed by real-time PCR targeting the type III secretion system genes of *B. pseudomallei* (*6*), and the isolate was later sequenced for a study of geographic dissemination of *B. pseudomallei* (*7*). After this identification, we conducted a retrospective analysis of archived blood culture isolates collected during 1994–2012 to investigate possible missed cases of invasive *B. pseudomallei* infection. 

## The Study

During 1994–1998, blood culture was performed on all febrile patients admitted to the pediatric wards at KCH. Since 1998, all pediatric patients <15 years of age admitted, except those having trauma, burns, or elective surgery, have had blood samples drawn for culture. Surveillance for patients >15 years of age began in 2007; blood samples are drawn at admission for cultures on patients meeting clinical criteria for possible invasive bacterial disease. Since 2002, hospitalization events have been linked to the Kilifi Health and Demographic Surveillance System (KHDSS), which monitors the population of ≈280,000 over an area of 891 km^2^ (*8*). Informed consent is obtained from all patients participating in the surveillance, including for storage of isolates and future use of clinical data.

Blood samples for bacterial cultures were collected in BACTEC Peds Plus or BACTEC Plus Aerobic/F bottles (Becton Dickinson, https://www.bd.com) and incubated on a BACTEC FX 9050 Automated Blood Culture instrument (Becton Dickinson). Nonfastidious, oxidase-positive, gram-negative bacilli were identified by using API 20NE test kits (bioMérieux, https://www.biomerieux.com). We reviewed all gentamicin-resistant, glucose-nonfermenting, gram-negative rods, with the exception of *Pseudomonas aeruginosa*, even if the API 20NE identification was acceptable, to account for difficulties in speciating *Burkholderia* spp. with biochemical methods.

A total of 86,582 patients <15 years of age were admitted during 1994–2012 and 18,864 patients ≥15 years of age during 2007–2012. Surveillance identified 33 gentamicin-resistant, glucose-nonfermenting bacilli in 14,235 positive blood cultures from patients <15 years of age and 5 gentamicin-resistant, glucose-nonfermenting bacilli in 705 positive blood cultures from patients ≥15 years of age (Figure). We retrieved all 38 isolates from storage for PCR, which we performed using published primer and probe sequences (*6*).

**Figure:**
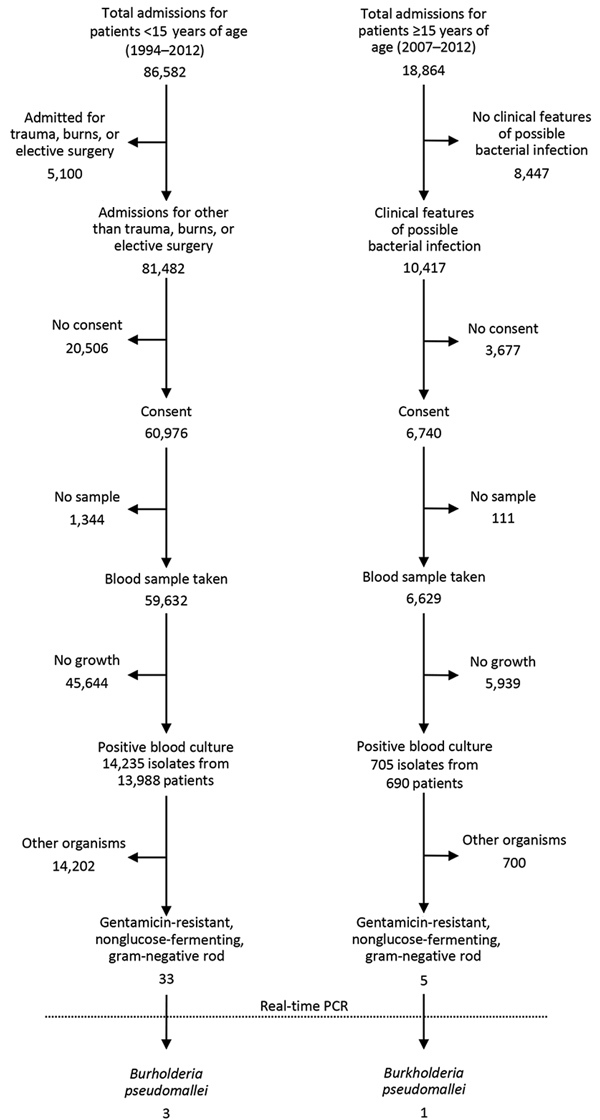
Identification of gentamicin-resistant, glucose-nonfermenting bacilli and *Burkholderia pseudomallei* from isolates collected from patients at Kilifi County Hospital, Kilifi, Kenya, 1994–2012.

We identified 4 isolates as *B. pseudomallei* by PCR, including the index isolate from 2010 (Table 1; Appendix). One isolate was previously identified as *B*. *cepacia,* and 2 were previously labeled as *Pseudomonas* species. We identified a fifth *B. pseudomallei* case in July 2014 in a 68-year-old female patient with diabetes mellitus and bilateral cervical abscesses (Table 1; Appendix). Blood culture results were negative, but aspirated pus grew *B. pseudomallei*, identified by API 20NE and confirmed by PCR. 

**Table 1:** Clinical summary of patients with positive *Burkholderia pseudomallei* isolates, Kilifi, Kenya, 2002–2014*

Year	Age/sex	Clinical features	Risk factors	Diagnosis†	Culture source	Antimicrobial sensitivity					Days in hospital	Outcome
						AMC	STX	TET	CAZ	IMI		
2002	8 d/M	Fever, jaundice, respiratory distress	None identified	Neonatal sepsis	Blood	S	S	S	S	S	3	Died
2008	7 d/M	Respiratory distress	None identified	Severe pneumonia, neonatal sepsis	Blood	S	S	S	S	S	3	Survived
2010	3 y/F	Fever, respiratory distress	Near-drowning	Severe pneumonia, septic shock	Blood	S	S	S	S	S	6	Died
2011	52 y/M	Persistent fever and night sweats of unknown duration	None identified	Acute renal failure, meningitis	Blood	S	S	S	S	S	5	Died
2014	68 y/F	Fever, bilateral cervical neck swellings	Diabetes mellitus	Diabetes, cervical lymphadenitis	Pus swab	S	S	S	S	S	40	Survived

*AMC, amoxicillin/clavulanic acid; CAZ, ceftazidime; IMI, imipenem; S, susceptible; STX, trimethoprim/sulfamethoxazole; TET, tetracycline. †Diagnosis at time of admission.

None of the case-patients had any history of travel outside Kilifi County. Three died during the course of their admission. No further information is available for the 2 case-patients who survived because they were not residents of the area surveyed by KHDSS.

To estimate the incidence of melioidosis bloodstream infection, we divided the number of invasive *B. pseudomallei* cases among KHDSS residents by the sum of the annual midyear population counts during 2002–2012 for those <15 years of age and during 2007–2012 for those >15 years of age. We also adjusted for the sensitivity of the surveillance to account for the proportion of patients not consenting to the surveillance study and those who did not have a blood culture drawn. For the period before 2002, we extrapolated age-specific population estimates by using a log-linear model of age-specific population data based on subsequent enumerations. The estimated incidence was 1.3 cases/1 million person-years of observation for those <15 years of age and 2 cases/1 million person-years of observation for those ≥15 years of age (Table 2).

**Table 2:** Incidence of melioidosis in Kilifi County Hospital, Kilifi, Kenya, 1994–2012*

Patient age group	No. cases	No. case-patients residing in KHDSS area	Study period	Person-years of observation	Crude incidence†** (95% CI)**	Adjusted incidence† (95% CI)
<15 y	3	2	1994–2012	2,026,781	1.0 (0.12–3.56)	1.3 (0.17–5.17)
≥15 y	1	1	2007–2012	782,373	1.3 (0.03–7.1)	2.0 (0.08–15.6)
Overall	4	3	NA	2,809,154	1.1 (0.22–3.12)	1.5 (0.35–5.0)

*KHDSS, Kilifi Health and Demographic Surveillance System; NA, not applicable. †Incidence per 10^6^ person-years of observation, adjusted for nonconsenters and missing blood cultures among eligible consenters.

## Conclusions

We identified 5 cases of melioidosis from a single surveillance site in Kenya. Despite reports suggesting that melioidosis is endemic but underdetected in the region (*5*), we demonstrated low incidence in this part of Kenya. Even so, *B. pseudomallei* has emerged as an underdiagnosed cause of sepsis in Kilifi County. The empirical treatment used for sepsis, ampicillin and gentamicin, does not cover *B. pseudomallei*. The lack of pathognomonic clinical features makes it difficult to detect melioidosis clinically, especially in areas to which the disease is not endemic. In the series we report, 2 case-patients died before receiving definitive treatment, and only 1 case-patient received antimicrobial drugs recommended to treat melioidosis.

The integrated, population-based bacterial surveillance system in Kilifi County provides a unique opportunity to estimate incidence. Routine blood culture sampling of all admitted patients <15 years of age and eligible patients >15 years of age eliminates reliance on clinical suspicion for bacteremic melioidosis. The use of molecular methods on isolates suspected to be *B. pseudomallei* will probably enhance case detection because *B. pseudomallei* is commonly misidentified or unidentified by culture (*9*). Only 2 isolates in our study were identified by using standard techniques, despite the reported good discriminatory performance of API 20NE in distinguishing *B. pseudomallei* and *B. cepacia* (*10*).

Our reported incidence rates might still be underestimated. Our data do not account for KHDSS residents who do not go to KCH. For example, ≈64% of deaths in children <5 years of age in the KHDSS area occur at home or in other healthcare facilities (*8*). Furthermore, as demonstrated by the fifth case, the incidence of nonbacteremic infection might be higher because non–blood culture samples are not systematically collected. Only 50%–75% of patients with melioidosis are bacteremic (*11*), and culture has an estimated sensitivity of 60.2% for melioidosis (*12*). In addition, our screening method excluded gentamicin-susceptible isolates. If gentamicin-susceptible *B. pseudomallei* is as common in Kenya as reported in other areas (*13*), additional surveillance that includes these organisms could increase the reported incidence rates. Finally, melioidosis often is unevenly distributed within endemic areas, as noted in Thailand (*14*). Despite these factors, our results suggest that, although *B. pseudomallei* is present in tropical Africa, the incidence of invasive melioidosis is surprisingly low.

The differences in disease incidence in Africa and Asia are striking. Host factors, such as diabetes mellitus, might contribute, but environmental factors and agricultural practices, such as rice farming, are probably more important in permitting exposure to and environmental persistence and proliferation of the organism. Nonetheless, Kenya has been identified as environmentally suitable for *B. pseudomallei* because of its soil type, agricultural practices, and rainfall (*1*). Our study demonstrates the presence of *B. pseudomallei* in Kenya. Changes in climate and agricultural practices might lead to future increases in melioidosis, and ongoing surveillance is necessary.

Appendix Case summaries of patients with positive *Burkholderia pseudomallei* isolates, Kilifi, Kenya, 2002–2014.
