# Supplementary material for: Population-Based Estimate of Melioidosis, Kenya
Source: Emerg Infect Dis. 2019 May;25(5):984–7. doi: 10.3201/eid2505.180545 (PMC6478202; doi:10.3201/eid2505.180545)
Supplement: Appendix — Case summaries of patients with positive Burkholderia pseudomallei isolates, Kilifi, Kenya, 2002–2014. [file 18-0545-Techapp-s1.pdf]

Article DOI: https://doi.org/10.3201/eid2505.180545

Population-Based Estimate of Melioidosis, Kenya

Appendix

Case Summaries of Patients with Melioidosis, Kilifi, Kenya

Patient 1. November 2002. An 8-day old male was admitted with a history of fever for 4 days and yellowness of eyes for 3 days. Mother reported normal breastfeeding. On examination he was febrile (temperature 39.0°C) and tachypneic (respiratory rate 84 cycles/min). He was pale, capillary refill time was prolonged, he had jaundice, chest wall indrawing and nasal flaring. The hemogram showed leukocytosis comprising mainly neutrophils, blood slide for malaria parasites was negative. Blood culture showed gram-negative rods that were later identified as *B. pseudomallei.* There was no growth from the cerebral spinal fluid culture. The patient died after 3 days of treatment in the ward. Records of antibiotic treatment were not available from the retrieved case record, but first line treatment for this condition in 2002 was penicillin and gentamicin (*1*).

Patient 2. June 2008. A 7-day-old male child was first seen with fever and a history of difficulty breathing for 3 days. The child had been born at home and his mother reported normal breastfeeding. On examination he was in respiratory distress and had chest wall indrawing, and a diagnosis of neonatal sepsis was made. There was no growth from the cerebrospinal fluid culture. He was treated empirically with ampicillin and gentamicin, and by day 3 had improved sufficiently to be discharged. Subsequently, a gentamicin-resistant nonglucose-fermenting gram-negative rod was isolated on day 5, which could not be identified further at that time. The child was not a resident of the KHDSS district, and no further follow-up information was available.

Patient 3. May 2010. A 3-year-old female child was admitted with a history of cough, difficulty breathing, and fever after a near drowning accident in a local seasonal river. She had been found unconscious with a distended abdomen. She later regained full consciousness but was not brought for treatment until 2 days later, when she developed respiratory symptoms. At

admission, she had a temperature of 38.9°C and a respiratory rate of 50 cycles/min; she had nasal flaring and chest wall indrawing. The white cell count showed leukocytosis (18.2×10^9 cells/L) and neutrophilia (73%) on the differential count. Her chest radiograph had patchy opacities and oxygen saturation measured by oximetry was 96% on room air. She had features of severe pneumonia, and was initially treated with ampicillin and gentamicin, which was changed to ceftriaxone after 48 hours because there was no improvement in the clinical condition. On day 5, cloxacillin and metronidazole were added to the treatment. On day 5, admission blood cultures grew gram-negative rods, which were identified as *Burkholderia pseudomallei (*API 20NE profile 1156576), susceptible to ceftazidime but resistant to gentamicin. The patient died on day 6 before any further changes to the treatment were effected.

Patient 4. October 2011. A 52-year-old man was seen with an acute respiratory illness after persistent fever and night sweats. The duration of symptoms was not recorded in the patient records we retrieved. He was febrile (temperature 38.4°C) at admission, but no other constituent clinical signs were elicited. Blood counts and white cell differential counts also were within normal ranges. Sputum was negative for acid-fast bacilli on direct microscopy. He was initially treated with penicillin and gentamicin. A blood culture taken at admission became positive on day 5 for gram-negative rods that were susceptible to ceftazidime and imipenem. The final identity of the organism was not fully determined at the time. The patient deteriorated and died on day 5. We could not retrieve any results showing renal function tests or cerebrospinal fluid studies from the patient's records although the discharge diagnosis on record was meningitis with acute renal failure.

Patient 5. July 2014. A 68-year-old female patient, with insulin-dependent diabetes mellitus, was seen with bilateral cervical abscesses. She had attended outpatient clinics 2 weeks previously for investigation of cervical lymphadenopathy suspected to be tuberculous lymphadenitis. A fine needle aspirate had been performed, the results of which were not available on request. At admission, she had hyperglycemia and bilateral neck abscesses that were incised and drained. The complete blood count, liver and renal function tests were normal. The pus aspirate grew *B. pseudomallei* (API 20NE Profile 1156573); blood culture results were negative. Abdominal ultrasound done was normal. She was managed on intravenous imipenem for 10 days and discharged on co-trimoxazole for the eradication phase regimen to continue for 3

months. She did not return for the follow up clinic appointments. She was not a resident of the KHDSS and no further follow-up information was available.

Reference

1. World Health Organization. Clinical guidelines for diagnosis and treatment of common conditions in Kenya. Nairobi: Ministry of Health; 2002 [cited 2018 Nov 01]. http://apps.who.int/medicinedocs/documents/s16427e/s16427e.pdf
